# An ELISA-based assay for determining haemagglutinin potency in egg, cell, or recombinant protein derived influenza vaccines

**DOI:** 10.3389/fimmu.2023.1147028

**Published:** 2023-03-22

**Authors:** Jesse Bodle, Kirsten Vandenberg, Karen Laurie, Ian G. Barr, Ying Zhang, Steven Rockman

**Affiliations:** ^1^ Vaccine Product Development, CSL Seqirus Ltd, Parkville, VIC, Australia; ^2^ Collaborating Centre for Reference and Research on Influenza, World Health Organisation, Melbourne, VIC, Australia; ^3^ Vaccine Product Development, CSL Seqirus Ltd, Holly Springs, NC, United States; ^4^ Department of Immunology and Microbiology, University of Melbourne, Parkville, VIC, Australia

**Keywords:** influenza potency assay, influenza vaccination, ELISA - enzyme-linked immunosorbent assay, influenza vaccine antigen, Monoclonal antibodies, immuno - assays

## Abstract

**Background:**

The current compendial assay for haemagglutinin antigen potency in influenza vaccine is the single radial immunodiffusion (SRID) which is time consuming and can lead to delays in release of vaccine. We previously described an alternate capture and detection enzyme linked immunoassay (ELISA) that utilizes sub-type specific, sub-clade cross-reactive monoclonal antibodies (mAbs) that are haemagglutination inhibiting (HAI) and correlate with SRID. The aim of this study is to determine the applicability of ELISA across current platforms for quantitation of seasonal quadrivalent vaccine.

**Methods:**

A single mAb capture and detection ELISA was employed to quantitate hemagglutinin (HA) derived from different vaccine platforms and host organisms and compared to SRID and a polyclonal antibody based ELISA.

**Results:**

We selected mAbs that displayed appropriate characteristics for a stability indicating potency assay which reacted to avian, insect and mammalian derived HA. Qualification of the homologous mAb assay against egg and cell derived HA demonstrated performance similar to that of the SRID however, superiority in sensitivity and specificity against strains from both influenza B/Victoria and B/Yamagata lineages. Analysis of drifted strains across multiple seasons demonstrated continued utility of this approach, reducing the need to develop reagents each season. With modification of the assay, we were able to accurately measure HA from different platforms and process stages using a single calibrated reference standard. We demonstrated the accuracy of ELISA when testing vaccine formulations containing selected adjuvants at standard and higher concentrations. Accelerated stability analysis indicated a strong correlation in the rate of degradation between the homologous mAb ELISA and SRID but not with ELISA utilizing polyclonal antisera. Further, we demonstrated specificity was restricted to the trimeric and oligomeric forms of HA but not monomeric HA.

**Conclusion:**

We believe this homologous mAb ELISA is a suitable replacement for the SRID compendial assay for HA antigen quantitation and stability assessment. Identification of suitable mAbs that are applicable across multiple vaccine platforms with extended sub-type reactivity across a number of influenza seasons, indicate that this assay has broad applicability, leading to earlier availability of seasonal and pandemic vaccines without frequent replacement of polyclonal antisera that is required with SRID.

## 1 Introduction

Vaccination is considered the best, most cost-effective defense against influenza virus infection (1). To provide optimum effectiveness, protein-based influenza vaccines should contain the matched antigen representing the surface proteins of a target pathogens that can elicit antibodies that facilitate viral neutralisation (1, 2). Developing vaccines to the influenza virus is particularly challenging since the surface antigens; haemagglutinin (HA) and neuraminidase (NA) evolve continuously resulting in the requirement for the vaccine to be updated regularly (2). This update, by the World Health Organisation (WHO), occurs bi-annually and is specific for each hemisphere (Northern/Southern) considering the strains that are circulating in each geographical location and providing a strain prototype recommendation for seasonal trivalent (sub-types: H1N1, H3N2 and Influenza B) and quadravalent formulations (sub-type: H1N1, H3N2, B Victoria and B Yamagata lineages) (2). Traditionally, most protein-based influenza vaccines were propagated in fertilized chicken eggs only, but more recently the host type and manufacturing platforms have expanded significantly in pursuit to deliver higher yielding and more effective vaccines (3–8). To prepare a vaccine, the recommended strain is formulated at an appropriate target amount of antigen to elicit an effective immune response based on the data derived from clinical trials. To measure antigen quantity, potency assays specific for the antigen are employed (2). In addition to quantifying antigen for formulation of vaccine drug substance and regulatory approved release to the market, a potency assay must measure antigen stability to ensure optimal potency of the vaccine over the shelf-life of the seasonal vaccine. The compendial influenza antigen potency assay; single radial immunodiffusion assay (SRID) has significant limitations (2, 9, 10) including the requirement of strain specific reagents that take 3-4 months to produce, relatively poor/variable limit of quantitation (LOQ) and sensitivity to formulation matrices such as adjuvants, which has led to a global interest in replacing this assay with an alternative potency assay. The ideal assay would accurately estimate the amount of antigen, reflect the immunogenicity of the antigen, have reagents that are easily and quickly obtainable, have broad cross-reactivity to avoid the need to update components as each new strain is introduced, be simple and cheap to perform and be applicable across various vaccine platforms.

We previously described a capture and detection enzyme linked immunoassay (ELISA) for egg based influenza vaccines that utilized sub-type specific monoclonal antibodies (mAbs) that were hemagglutination inhibiting and also stability indicating (when studied under stressed conditions including variation in temperature, pH and oxidative conditions) and also correlated with the standard SRID assay (11, 12). The mAbs developed for this assay were derived by immunizing mice with purified egg-derived influenza antigen which may make the assay platform specific and limited to egg-derived vaccines due to potential egg-adaption mutations that can occur when mammalian influenza viruses are propagated in embryonated hen’s eggs (13). In order to increase the breadth of application of this method to accommodate all the current global influenza vaccine industry, we developed this single mAb assay to be able to use in a range of antigen presentations, vaccine platforms and formulation matrices. The selections of cross platform mAbs described here were qualified to accurately determine the level of antigen regardless of presentation and potency values generated correlated well with the compendial SRID or expected target formulation values. In summary, our modified HA ELISA demonstrates all the qualities required for influenza vaccine formulation and GMP release of current licensed influenza vaccines with the added benefit of speed to implementation, robustness, sensitivity and simplicity and is a good candidate technology to replace the SRID method which was first developed for influenza vaccines back in the 1970’s (14).

## 2 Materials and methods

### 2.1 Monoclonal antibodies

Monoclonal antibodies were produced as previously described (15) using density gradient purified, whole inactivated or live influenza virus propagated in embryonated chicken eggs as immunogens. Antibodies were raised against the influenza strains A/Hiroshima/52/2005 (H3N2: HIR3.8G11.1B5), A/Singapore/37/2009 (H3N2; SIN3.9G11.2B6), A/Victoria/563/2010 (H3N2; AVIC142.2B5.1B9), A/Tasmania/11/2014 (H3N2; TAS160.7E8.1C4), A/Singapore/GP2050/2015 (H3N2; ASIN178.10G10.21F4), A/Brisbane/59/2007 (H1N1; ABR4.1D5.1B11), A/California/07/2009 (H1N1; CAL2.5C6.1E3), A/Singapore/GP1908/2015 (H1N1; SIN176.10B11.1E9), A/Victoria/2454/2019 (H1N1; TORA184. 9G2.21B11), A/Barheadedgoose/Qinguai/1A/2005 (H5N1; QIN2.8C10.1D10), B/Panama/45/1990 (Yamagata lineage; PM10.2D7.2F11.1G6), B/Wisconsin/1/2010 (Yamagata lineage; BWIS141.9B3.2D8B), B/Phuket/3073/2013 (Yamagata lineage; PHU168.10D10.1G8), B/Malaysia/2506/2004 (Victoria lineage MIA1.8E3.1E9), B/Brisbane/60/2008 (BBR3.10E10.1C3), B/Iowa/06/2017 (Victoria lineage IOWA181.6D10.17F5). The resulting hybridomas were screened and selected based on the method previously described with additional modifications (11) including screening of mAbs against panels of cell-derived reassortants (as well as egg derived), western analysis, mAb binning using Surface Plasmon Resonance (T200, Cytiva, MA, USA) to determine non-overlapping epitope binding, epitope mapping using both *in-silica* (DNASTAR V17.2.1 Novafold & Novadock, WI, USA) and experimental methodology (alanine substitution and overlapping peptide screening) and finally screening against HA antigen in a panel of vaccine matrices containing different levels of adjuvant, detergents and salts. Detection mAbs were conjugated with horse-radish peroxidase (HRP) according to manufacturers’ specifications (Lightning Link, Innova Biosciences, Cambridge UK).

### 2.2 SRID reagents

Sheep polyclonal anti-HA antibodies (pAbs) and strain matched inactivated influenza reference antigens (RAs), standardized for HA potency, were obtained from either the Therapeutic Goods Administration (TGA, Woden, ACT, Australia), the National Institute for Biological Standards and Control (NIBSC, Potters Bar, Hertfordshire, UK), or the Centre for Biological Evaluation and Research (CBER, Rockville, MD, USA). These reagents were raised against egg-derived strains: A/Singapore/INFIMH-16-0019/2016 (H3N2: TGA AS424 Ab and 2018/123B RA), A/Brisbane/1/2018 (H3N2: TGA AS428 Ab and 2018/125B RA), A/Egypt/NO3072/2015 (H5N1: TGA AS414 Ab and 2016/110B RA), A/New Caledonia/71/2014 (H3N2: TGA AS410 RA and 2015/107B Ag), A/Hong Kong/4801/2014 (H3N2: CBER H3-Ab-1611 Ab and H3-Ag-84 RA), A/California/7/2009 (H1N1: CBER H1-Ab-1304 Ab and H1-Ag-1104 RA), A/Singapore/GP1908/2015 (H1N1: TGA AS415 Ab and 2016/112B RA), A/Brisbane/02/2018 (H1N1: TGA AS430 Ab and 2019/127B RA), A/Kansas/14/2017 (H3N2: TGA AS433 Ab and 2019/128B RA), B/Maryland/15/2016 (BVIC: TGA AS422 Ab and 2018/122B RA), B/Brisbane/46/2016 (BVIC: TGA AS411 Ab and 2016/111B Ag), B/Phuket/3073/2013 (BYAM: TGA AS434 Ab and 2020/136B RA), B/Hubei-Wujiagang/158/2009 (BYAM: NIBSC 12/118 Ab and 12/106 RA) and B/Brisbane/60/2008 (BVIC: CBER B(v)-Ab-1108 Ab and B(v)-Ag-68 RA) and cell-derived strains A/Delaware/55/2019 (H1N1: TGA AS443 Ab and CBER H1-Ag-2017 RA), A/Idaho/07/2018 (H1N1: TGA AS430 Ab and CBER H1-Ag-1902 RA), A/Newcastle/82/2018 (H3N2: TGA AS437 Ab and NIBSC 19/204 RA), A/Indiana/08/2018 (H3N2 NIBSC 19/152 Ab and CBER H3-Ag-1904 RA), A/North Carolina/04/2016 (H3N2: NIBSC 18/108 Ab and CBER H3-Ag-1801 RA), B/Singapore/INFTT-16-0610/2016 (BYAM: CBER B(y)-Ab-1606 Ab and B(y)-Ag-1709 RA), B/Darwin/07/2019 (BVIC: TGA AS436 Ab and NIBSC 19/210 RA) and B/Iowa/06/2017 (BVIC: CBER B(v)-Ab-1807 and B(v)-Ag-1804 RA).

### 2.3 Influenza vaccine and recombinant HA

Monovalent influenza vaccine pools: Samples were collected for ELISA and SRID testing during the vaccine manufacturing process prior to vaccine formulation. For egg-derived virus (split virion) Afluria^®^; purified and concentrated virus propagated in embryonated chicken eggs, was inactivated using β-propiolactone, split using sodium taurodeoxycholate and processed by di-filtration to remove detergent and other impurities using proprietary methods (Seqirus Ltd, Melbourne, VIC, Australia). For cell-derived virus (surface antigen, subunit) Flucelvax^®^; purified and concentrated virus propagated in Madin Darby Canine Kidney (MDCK) cells, was inactivated using β-propiolactone, split using cetyltrimethylammonium bromide (CTAB) and processed to remove detergent and other impurities using proprietary methods (Seqirus Ltd, Holly Springs, NC, USA). For egg-derived virus (surface antigen, sub-unit) Fluad^®^; purified and concentrated virus propagated in embryonated chicken eggs, was inactivated using formalin, split using cetyltrimethylammonium bromide (CTAB) and processed to remove detergent and other impurities using proprietary methods (Seqirus Ltd, Liverpool, England, UK). Quadrivalent vaccines were formulated according to SRID potencies to contain no less than 15 micrograms HA per strain per dose (~30 mcg/mL), of WHO recommended strain candidates representing Influenza subtypes; H1N1, H3N2, B-Victoria lineage and B-Yamagata lineage. Recombinant HA expressed in HEK293 cells, E.coli (Creative Biomart Inc., NY, USA, catalogue numbers: HA-001 and HA-002 respectively) and baculovirus (Flublok^®^ Quadrivalent; lot number QFAA2152, Sanofi Ltd and cDNA recombinant HA, SinoBiological, Beijing, China; catalogue number 11085-vo8b) were purchased commercially.

### 2.4 ELISA optimization and intra-assay criteria

Optimisation of coating and detection for each ELISA was performed by titration of unconjugated and conjugated mAbs, respectively, with fixed concentrations of purified inactivated, split virion and subunit HA preparations (16). The optimum mAb and entry HA concentration was determined as the lowest concentration that yielded an appropriate 7 point sigmodal curve with well-defined upper (OD450nm ≥1.5) and lower (OD450nm ≤0.5) asymptotes (17–20). Monoclonals that are unable to achieve upper and lower asymptote criteria were considered not suitable for use in potency ELISA assays. Monoclonals with upper asymptotes of OD450nm ≥1.5, ≤2.0 were considered valid and suitable for use however they were also flagged for replacement later with a mAb from a new fusion.

### 2.5 HA potency by capture and detection ELISA

HA potency ELISA was conducted using homologous labelled and unlabelled mAbs for the capture and detection of native HA antigen, adapted from the method previously described (11), with the following modifications: Microtitre plates were coated with 100 µl/well of unconjugated mAbs diluted to an optimised concentration between 0.4 – 2 µg/mL in Dulbecco’s PBS w/o Ca, Mg (ThermoFisher, MA, USA) and incubated at 2-8°C overnight. Samples and RAs were incubated with zwittergent 3-14 (Calbiochem, Darmstadt, Germany) at a final concentration of 0.5% (w/v) per 30 µg of HA for 1 hour (RT) before two-fold serial dilutions in assay diluent (AsureQuality, Victoria, Australia) to produce 7 point curves targeting pre-determined HA concentrations between 4 – 10 µg/mL. Plates were incubated with 100 µL/well of HRP-conjugated mAb diluted to optimised concentrations between 0.16 – 2 µg/mL. The HA concentrations of samples were determined by 4-parameter logistical fit (4PL) with parallel line regression analysis (Softmax Pro GxP v7.0, Molecular Devices, CA, USA) against process matched split virion or subunit HA RAs, calibrated independently using biophysical methods: namely SDS-PAGE densitometry (21) (for egg-derived HA) and isotope dilution mass spectrometry (IDMS) (22) (for cell-derived, baculovirus derived and recombinant protein HA). Internally calibrated ELISA RAs were tested in SRIDs against official ERL calibrated SRID reagents to verify accuracy. Sample concentration was calculated based on its relative potency (RP) to that of the reference antigen.

### 2.6 Antigen directly coated ELISA

Microtiter plates were coated directly with 100 µl/well of samples containing HA antigen at a target concentration of 5µg/mL in Dulbecco’s PBS w/o Ca, Mg (ThermoFisher, MA, USA) and incubated at 2-8°C overnight. Unconjugated mAb diluted to an optimised concentration between 0.4 – 2 µg/mL was then added neat or titrated by two-fold steps in assay diluent against coated HA antigen. mAb reactivity was then assessed by the addition of an HRP conjugated goat anti mouse antibody added at manufacturers recommended concentration (ELITechGroup KPL, Belgium, Germany). The assay was completed as described (11).

### 2.7 HA potency by SRID

SRID assays were performed as previously described (12). Briefly, RA and test antigen materials were diluted 1:1, 2:3 and 1:3 (v/v) in PBS containing 1% (w/v) Zwittergent solution (Calbiochem, Darmstadt, Germany), and added to duplicate wells of agarose gels containing polyclonal antiserum listed (SRID reagents). Gels were incubated between 18 - 24 hours in humidified chambers, dried onto glass plates, and stained with Coomassie brilliant blue R-250 (Sigma, California, USA). Circular zones of antigen-antibody precipitation were measured, and HA concentration was calculated by the parallel line bioassay method in comparison to purified inactivated, whole virion reference antigen (23), and test validity was confirmed using a ‘g’ test (g ≤ 0.061) (24).

### 2.8 Qualification of ELISA and SRID methods

Strain specific qualification was conducted with optimised reagents in both ELISA and SRID assays in accordance with ICH guidelines (25). Repeatability was defined as four replicate results of at least five concentrations of the sample preparations with Coefficient Variations (CVs) of ≤ 15% for all concentrations. Intermediate Precision (IP) was defined as a minimum of six independent potencies obtained by no less than two analysts over two or more days with CVs ≤ 18%. Range was defined by the lowest and highest relative potencies against the reference antigen which produced linear, accurate and precise results. Minimum accepted range was between 80% to 120% of the sample concentration. The assay was considered accurate if the percentage recovery of sample preparations was within 20% (80-120%) of the expected concentration. The Limit of Detection (LOD) was defined as the lowest theoretical concentration of HA based on dilution factor, that could be detected above background plus 3 standard deviations. The Limit of Quantification (LOQ) was defined as the highest dilution of reference antigen that could be accurately quantitated within an accuracy (observed *vs* expected) greater than 75% and less than 125%. The specificity of the assay was defined as the antigen being detected by homologous antisera only, or, if cross-reactivity occurs (SRID only), the assay will be considered specific if antigen meets criteria for accuracy.

### 2.9 Hemagglutination inhibition assay

Monoclonals were screened by a standard hemagglutination inhibition assay. Briefly, two-fold serial dilutions of mAb (25 μl/well) starting at a concentration of 2μg/mL mAb were mixed with 4 HA units of virus (25 μl/well) in 96-well microtiter plates and incubated for 1 hour at RT. A 1% hematocrit formulation of red blood cell (fowl or guinea pig) in PBS- was added (50 μl) and the plate was incubated for a further 1 hour at RT. The HAI HI titer was determined as the reciprocal of the highest mAb dilution that inhibited hemagglutination.

### 2.10 Size exclusion ultra high performance liquid chromatography

An ultra-high-performance liquid chromatography system (UltiMate 3000 BioRS, Thermo Scientific) coupled to an FLD-3400RS fluorescence detector (Thermo Scientific) was used for performing the analytical-scale SE-UHPLC fractionation. Flucelvax monovalent drug substance (3mg) was applied to a Superose 6 Increase 10/300 GL column, (Cytiva, Cat# 29-0915-96) equilibrated in running buffer (0.37 g/L monobasic potassium phosphate, 1.29 g/L disodium phosphate, 0.1 g/L magnesium chloride, 0.2 g/L potassium chloride and 8.0 g/L sodium chloride, pH 7.2) and run through the column at a rate of 0.12 mL/min (column temperature 30°C). Antigen peaks were collected as 16 x 1mL fractions with an AKTA pure chromatography system (Cytiva) and pooled to represent HA species: high molecular weight (MW) HA oligomers A (fractions 1-5), high MW HA oligomers B (fractions 9 and 10), HA trimer (fraction 12) and HA monomer (fraction 15). In-line fluorescence data (FLD excitation 280nm, emission 345nm) was assessed using Chromeleon 7.2 software package. The molecular weight of SE-UHPLC separated HA species was determined using gel filtration standards (Bio-RAD, cat# 151-1901). The 4 fractions including Monovalent control antigens were normalized by total protein nitrogen before being assayed *via* SRID and ELISA methods.

## 3 Results

### 3.1 Longevity of cross-reactive monoclonal reagents demonstrate versatility across drifted strains over multiple seasons

The utility of ELISA was assessed against a total of 53 H3N2 strains (45 egg-derived, 8 cell-derived) representing 26 genetic subclades (**Table 1**). Over this period of 19 years, which covered 36 vaccine seasons from both the Northern and Southern Hemispheres (NH and SH), new SRID reagents were required to be produced on a total of 23 occasions (egg and cell vaccine inclusive). Across this same period of time, ELISA was established for all vaccine candidate strains utilizing 5 mAbs (clones HIR3.8G11, SIN3.9G11, VIC142.2B5, TAS160.7E8 and SIN170.10G10). The HIR3.8G11 reacted (sufficient for qualified ELISA) with all pre clade 1 viruses isolated between 2003-2008. Clone SIN3.9G11 reacted (sufficient for qualified ELISA) with 1/9 pre clade 1viruses, clade 1 (2/2), clade 6 (1/1), clade 5/6 (1/1), and weakly (not suitable for qualified) against 12/40 viruses of the 3C sub-clade lineage. Clone VIC142.2B5 had good reactivity (suitable for qualified ELISA) against clade 1(2/2), clade 5/6 (1/1) and sub-clades 3C and 3C.1 viruses, however, reacted weakly or not at all against all successive viral strains emerging after sub-clade 3C.1 (post 2012). Clone TAS160.7E8 reacted well (sufficient for qualified ELISA) with most viruses of sub-clade 3C.1, 3C.3a, 3C.2a and 3C.3a1 however, reacted poorly, inconsistently or not at all against virus from sub-clades: 3C.2a1, 3C.2a3 and successive viral strains emerging. Finally, clone SIN178.10G10 reacted with all viruses, beginning at clade 1, between 2009 and continues to be in-use today (2023). These H3N2 mAbs were also tested against representative strains of H1N1 sub-type, B/Victoria and B/Yamagata lineage demonstrating H3N2 sub-type specificity. Similar observations were made when drifted strains from the 3 other sub-type/lineages of the seasonal Afluria™ and Flucelvax™ vaccine were screened. For the H1N1 sub-type (**Table 2**), 4 mAbs (ABR4.1D5, CAL2.5C6, SIN176.10B11 and TORA184.9G2) gave continuous coverage to 33 H1N1 strains spanning 14 clades/sub-clades between the years 2006 – 2022, representing 16 NH and 16 SH campaigns in total. Three mAbs (CAL2.5C6, SIN176.10B11 and TORA184.9G2) gave coverage to 30 circulating strains, including the 2009 H1N1 pandemic strain: A/California/07/2009 and all seasonal H1N1 vaccine strains post 2009. Two mAbs (BBR3.10E10 and IOWA181.6D10) were sufficient to cover all B Victoria lineage strains tested between 2007 – 2022 vaccine seasons (n=14), including the 8 strains used in both Afluria™ and Flucelvax™ vaccines over the past 15 years (**Table 3**). Strain: B/Hong Kong/259/2010, which was included in the 2016 and 2017 Flucelvax™ formulation was not screened in this study, however its HA sequence shared >99% homology (MegAlign Pro, DNASTAR V17.2.1, WI, USA) with prototype virus B/Brisbane/60/2008 thus would likely cross-react with both mAbs. Three mAbs (PM10.2D7, BWIS141.10F9 and PHU168.10D10) were cross-reactive against strains of the B/Yamagata lineage spanning 34 years, from the time Influenza B diverged into two lineages (**Table 4**). Notably, two monoclonal antibodies (BWIS141.10F9 and BPHU168.10D10) were able to be used for qualified potency ELISAs against every B Yamagata vaccine strain included in both Afluria™ and Flucelvax™ vaccines over the last 16 years. Overall, the average level of cross-reactivity with drifted strains was considered; 4.0 years (4 mAbs, 16 year period) for H1N1, 4.8 years (4 mAbs, 19 year period) for H3N2, 4.7 years (3 mAbs, 14 year period) for B Victoria and 14 years (2 mAbs, 28 years) for B Yamagata.

**Table 1:** Cross reactivity of monoclonal antibody (mAb) panel specific to sub-type H3N2 screened against egg derived (E) and cell derived (C) vaccine antigen.

Clade/ Sub-Clade	Strain	Sub-type	H3N2					H1N1	BVIC	BYAM
		Fusion ID	HIR3	SIN3	VIC142	TAS160	SIN178	CAL2	BBR3	BWIS
		Parental Line	8G11	9G11	2B5	7E8	10G10	5C6	10E10	10F9
Unassigned	A/Wyoming/3/03**^E^**		**+++**	–	–	–	–	–	–	–
Unassigned	A/Wellington/1/04 **^E,V^**		**[+++]**	–	–	–	–	–	–	–
Unassigned	A/Hiroshima/52/05 **^E,V^**		**[+++]**	–	–	–	–	–	–	–
Unassigned	A/Wisconsin/67/05 **^E,V^**		**[+++]**	–	–	–	–	–	–	–
Unassigned	A/Nepal/921/06 **^E^**		**+++**	–	–	–	–	–	–	–
Unassigned	A/Brisbane/10/07 **^E,V^**		**[+++]**	–	–	–	–	–	–	–
Unassigned	A/Uruguay/716/07 **^E,V^**		**[+++]**	–	–	–	–	–	–	–
Unassigned	A/Brisbane/24/08 **^E^**		**+**	–	–	–	–	–	–	–
1	A/Wisconsin/15/09 **^E,V^**		–	**[+++]**	**+++**	**+++**	**+++**	–	–	–
1	A/Victoria/210/09 **^E,V^**		–	**[+++]**	**+++**	**+++**	**++**	–	–	–
Unassigned	A/Victoria/208/09 **^E^**		–	**+++**	–	–	**++**	–	–	–
6	A/Alaska/05/10 **^E^**		–	**+++**	–	–	**++**	–	–	–
5/6	A/Victoria/563/10 **^E^**		–	**+++**	**+++**	–	**++**	–	–	–
3C	A/Victoria/361/2011 **^E,V^**		–	**++**	**[+++]**	**++**	**+++**	–	–	–
3C.1	A/Texas/50/2012 **^E,V^**		–	**++**	**[+++]**	**+++**	**+++**	–	–	–
3C.3a	A/South Australia/55/2014 **^E,V^**		–	–	–	**[+++]**	**+++**	–	–	–
3C.2a	A/New Caledonia/71/14 **^E^**		–	–	–	**+++**	**+++**	–	–	–
3C.2a	A/Hong Kong/4801/2014 **^E,V^**		–	**+**	–	**[+++]**	**+++**	–	–	–
3C.2a1a	A/Norway/3806/2016 **^E^**		–	**+**	–	**++**	**+++**	–	–	–
3C.2a1	A/Brisbane/285/2016 **^E^**		–	**+**	–	**++**	**+++**	–	–	–
3C.2a2	A/Brisbane/321/2016 **^E^**		–	**+**	–	**+++**	**+++**	–	–	–
3C.2a1	A/Singapore/INFIMH-16-0019/2016 **^E,V^**		–	**++**	–	**++**	**[+++]**	–	–	–
3C.2a1	A/Singapore/GP2646/2016 **^E^**		–	–	–	–	**+++**	–	–	–
3C.2a3	A/Singapore/TT13774/2016 **^E^**		–	–	–	–	**+++**	–	–	–
3C.2a	A/North Carolina/04/2016 **^C,V^**		–	–	–	**+**	**[+++]**	–	–	–
3C.2a1b.1	A/Hong Kong/2286/2017 **^E^**		–	**+**	–	**+++**	**+++**	–	–	–
3C.2a1b.1	A/Victoria/624/2017 **^E^**		–	**+**	–	**+++**	**++**	–	–	–
3C.2a2	A/Switzerland/8060/2017 **^E,V^**		–	**+**	–	**++**	**[+++]**	–	–	–
3C.2a1	A/Greece/4/2017 **^E^**		–	**+**	–	**++**	**+++**	–	–	–
3C.3a1	A/Kansas/14/2017 **^E,V^**		–	–	–	**[+++]**	**+++**	–	–	–
3C.2a1b.1	A/Netherlands/10260/2018 **^E^**		–	–	**+**	**+++**	**++**	–	–	–
3C.3a	A/Brisbane/34/2018 **^E^**		–	**+**	–	**+++**	**++**	–	–	–
3C.2a1b.2	A/Newcastle/82/2018 **^C,V^**		–	–	–	–	**[+++]**	–	–	–
3C.2a1b.2	A/Newcastle/104/2018 **^E^**		–	–	–	**++**	**+++**	–	–	–
3C.3a1	A/Indiana/08/2018 **^C,V^**		–	–	–	**++**	**[+++]**	–	–	–
3C.2a1b.2	A/South Australia/34/2019 **^E,V^**		–	–	**+**	**[+++]**	**++**	–	–	–
3C.2a1b 1b	A/Hong Kong/2671/2019 **^E,V^**		–	–	–	–	**[+++]**	–	–	–
3C.2a1b 1a	A/Paris/2554/2019 **^E^**		–	–	–	–	**+++**	–	–	–
3C.2a1b 1b	A/Pennsylvania/1026/2019 **^E^**		–	–	–	**+++**	**+++**	–	–	–
3C.2a1b 1b	A/Pennsylvania/1025/2019 **^E^**		–	–	–	–	**+++**	–	–	–
3C.2a1b 1b	A/Delaware/39/2019 **^C,V^**		–	–	–	–	**[+++]**	–	–	–
3C.2a1b 2a	A/South Australia/474/2019 **^E^**		–	–	–	–	**++**	–	–	–
3C.2a1b 1a	A/Perth/20/2020 **^E^**		–	–	–	–	**++**	–	–	–
3C.2a1b 2a.1	A/Tasmania/503/2020**^C,V^**		–	–	–	+++	**[+++]**	–	–	–
3C.2a1b 2a.1	A/Tasmania/503/2020 **^E^**		–	–	–	–	**++**	–	–	–
3C.2a1b 2a.1	A/Cambodia/e0826360/2020 **^E,V^**		–	–	–	**++**	**[+++]**	–	–	–
3C.2a1b 2a.2	A/Bangladesh/911009/2020 **^E^**		–	–	–	–	**+++**	–	–	–
3C.2a1b 2a.2	A/Bangladesh/3005/2020 **^E^**		–	–	–	–	**+++**	–	–	–
3C.2a1b 2a.2	A/Darwin/9/2021 **^E,V^**		–	–	–	–	**[+++]**	–	–	–
3C.2a1b 2a.2	A/Darwin/11/2021 **^C,V^**		–	–	–	–	**[+++]**	–	–	–
3C.2a1b 2a.2	A/Darwin/6/2021 **^E^**		–	–	–	–	**+++**	–	–	–
3C.2a1b 2a.2	A/Victoria/6/2022**^C^**		–	–	–	–	**+++**	–	–	–
3C.2a1b 2a.2	A/Victoria/12/2022**^C^**		–	–	–	–	**+++**	–	–	–
Negative Controls	H1N1	A/Singapore/GP1908/2015 **^E,V^**	–	–	–	–	–	**++**	–	–
	BVIC	B/Brisbane/60/08 **^E,V^**	–	–	–	–	–	–	**[+++]**	–
	BYAM	B/Phuket/3073/2013 **^E,V^**	–	–	–	–	–	–	–	**[+++]**

The quality of mAb reactivity was graded and represented as following: reactivity ≥ 3 SDs above mean background but not sufficient to support potency ELISA (+), reactivity sufficient to support potency ELISA however at edge of optimal conditions outlined in methods section (++) and reactivity optimal to support a qualified potency ELISA (+++). Strains that were not screened against candidate mAbs (-). Strains selected and formulated into the commercially released Afluria™ and Flucelvax™ vaccines are identified (V). All mAbs assessed in HAI were able to neutralize both cell and egg derived viruses. Vaccine candidate strains were screened against mAbs incorporated in qualified ([+++]) and/or un-qualified (+++) ELISA assays.

**Table 2:** Cross reactivity of monoclonal antibody (mAb) panel specific to sub-type H1N1 screened against egg derived (E) and cell derived (C) vaccine antigen.

Pre or Post 2009 H1N1 Pandemic	Clade/ Sub-Clade	Strain	Sub-type:	H1N1				H3N2	BVIC	BYAM
			Fusion ID	ABR4	CAL2	SIN176	TORA184	SIN178	BBR3	BWIS
			Parental Line	1D5	5C6	10B11	9G2	10G10	10E10	10F9
Pre 2009 Pandemic	2A	A/Solomon Island/3/2006**^E,V^**		[+++]	–	–	–	–	–	–
	2A	A/Fukushima/141/2006**^E^**		+++	–	–	–	–	–	–
	2B	A/Brisbane/59/2007**^E,V^**		[+++]	–	–	–	–	–	–
Post 2009 Pandemic	1	A/California/07/2009**^E,V^**		–	[+++]	–	+++	–	–	–
	4	A/Brisbane/10/2010**^E^**		–	+++	–	–	–	–	–
	7	A/Victoria/502/2010**^E^**		–	+++	–	–	–	–	–
	7	A/Brisbane/70/2011**^E^**		–	+++	–	–	–	–	–
	6B.1	A/Singapore/GP1911/2015**^E^**		–	+++	+	–	–	–	–
	6B.1	A/Michigan/45/2015**^E^**		–	++	+++	+++	–	–	–
	6B.1	A/Singapore/GP1908/2015**^E,V^**		–	++	[+++]	+++	–	–	–
	6B.1	A/Singapore/GP1908/2015**^C,V^**		–	–	[+++]	+++	–	–	–
	6B.1A.1	A/Brisbane/02/2018**^E,V^**		–	+++	[+++]	–	–	–	–
	6B.1A.5a	A/Darwin/6/2018**^E,V^**		–	+++	[+++]	–	–	–	–
	6B.1A.5	A/Darwin/122/2018**^E^**		–	+++	+++	–	–	–	–
	6B.1A.3	A/Idaho/07/2018**^C,V^**		–	–	[+++]	–	–	–	–
	6B.1A.2	A/Canberra/13/2019**^E^**		–	+++	–	–	–	–	–
	6B.1A.5a.1	A/Nebraska/14/2019**^E^**		–	–	++	–	–	–	–
	6B.1A.5a.1	A/Victoria/2455/2019**^E^**		–	++	+++	–	–	–	–
	6B.1A.5a.1	A/Victoria/2454/2019**^E^**		–	+++	+++	+++	–	–	–
	6B.1A.5a.2	A/Victoria/2570/2019 **^E^**		–	+++	–	++	–	–	–
	6B.1A.5a.2	A/Delaware/55/2019**^C,V^**		–	–	–	[+++]	–	–	–
	6B.1A.5a.1	A/Nebraska/14/2019**^C,V^**		–	–	–	[+++]	–	–	–
	6B.1A.5a.2	A/Illinois/02/2020**^C,V^**		–	–	–	[+++]	–	–	–
	6B.1A.5a.2	A/Victoria/3/2020**^E^**		–	+++	–	++	–	–	–
	6B.1A.5a.2	A/Victoria/1/2020**^E^**		–	+++	–	++	–	–	–
	6B.1A.5a.2	A/Tasmania/509/2020**^E^**		–	–	–	++	–	–	–
	6B.1A.5a.2	A/Darwin/118/2020**^C^**		–	–	–	+++	–	–	–
	6B.1A.5a.2	A/Victoria/1/2020**^E^**		–	+++	–	+++	–	–	–
	6B.1A.5a.2	A/Darwin/116/2020**^C^**		–	–	–	+++	–	–	–
	6B.1A.5a.1	A/Canberra/40/2020**^C^**		–	–	–	++	–	–	–
	6B.1A.5a.2	A/Sydney/173/2022**^C^**		–	–	–	+++	–	–	–
	6B.1A.5a.1	A/Brisbane/50/2022**^C^**		–	–	–	+++	–	–	–
	6B.1A.5A.2	A/Sydney/5/2022**^C,V^**		–	–	–	[+++]	–	–	–
Negative Controls	H3N2	A/Victoria/361/2011**^E,V^**		–	–	–	–	[+++]	–	–
	BVIC	B/Brisbane/60/08**^E,V^**		–	–	–	–	–	[+++]	–
	BYAM	B/Phuket/3073/2013**^E,V^**		–	–	–	–	–	–	[+++]

The quality of mAb reactivity was graded and represented as following: reactivity ≥ 3 SDs above mean background but not sufficient to support potency ELISA (+), reactivity sufficient to support potency ELISA however at edge of optimal conditions outlined in methods section (++) and reactivity optimal to support a qualified potency ELISA (+++). Strains that were not screened against candidate mAbs (-). Strains selected and formulated into the commercially released Afluria™ and Flucelvax™ vaccines are identified (V). All mAbs assessed in HAI were able to neutralize both cell and egg derived viruses. Vaccine candidate strains were screened against mAbs incorporated in qualified ([+++]) and/or un-qualified (+++) ELISA assays.

**Table 3:** Cross reactivity of monoclonal antibody (mAb) panel specific to influenza B Victoria lineage screened against egg derived (E) and cell derived (C) vaccine antigen.

Clade/ Sub-Clade	Strain	Lineage	B VIC			H1N1	H3N2	BYAM
		Strain	MIA1	BBR3	IOWA181	CAL2	SIN3	BWIS
		Parental Line	8E3	10E10	6D10	5C6	10G10	10F9
1	B/Malaysia/2506/2007**^E,V^**		[+++]	+	++	–	–	–
2	B/Brisbane/46/2008**^E^**		–	+++	+	–	–	–
2	B/Brisbane/60/2008E,**^C,V^**		–	[+++]	+++	–	–	–
V1A	B/Brisbane/46/2015**^E^**		–	+++	+++	–	–	–
V1A	B/Maryland/15/2016**^E^**		–	+++	+++	–	–	–
V1A.1	B/Colorado/06/2017**^E,V^**		–	[+++]	+++	–	–	–
V1A.1	B/Iowa/06/2017**^C,V^**		–	–	[+++]	–	–	–
V1A.3	B/Victoria/705/2018**^E^**		–	+++	+++	–	–	–
V1A.3	B/Washington/02/2019**^E,V^**		–	–	[+++]	–	–	–
V1A.3	B/Darwin/7/2019**^C,V^**		–	–	[+++]	–	–	–
V1A.3a.2	B/Stockholm/10/2020**^E^**		–	–	++	–	–	–
V1A.3a.2	B/Austria/1359417/2021**^E,V^**		–	–	[+++]	–	–	–
V1A.3a.2	B/Michigan/01/2021**^E^**		–	–	+++	–	–	–
V1A.3a.2	B/Singapore/WUH4618/2021**^C,V^**		–	–	[+++]	–	–	–
Negative Controls	H3N2: A/Victoria/361/2011**^E,V^**		–	–	–	–	[+++]	–
	H1N1: A/Singapore/GP1908/2015**^E,V^**		–	–	–	++	–	–
	BYAM: B/Phuket/3073/2013**^E,V^**		–	–	–	–	–	[+++]

The quality of mAb reactivity was graded and represented as following: Reactivity ≥ 3 SDs above mean background but not sufficient to support potency ELISA (+), reactivity sufficient to support potency ELISA however at edge of optimal conditions outlined in methods section (++) and reactivity optimal to support a qualified potency ELISA (+++). Strains that were not screened against candidate mAbs (-). Strains selected and formulated into the commercially released Afluria™ and Flucelvax™ vaccines are identified (V). All mAbs assessed in HAI were able to neutralize both cell and egg derived viruses. Vaccine candidate strains were screened against mAbs incorporated in qualified ([+++]) and/or un-qualified (+++) ELISA assays.

**Table 4:** Cross reactivity of monoclonal antibody (mAb) panel specific to influenza B Yamagata lineage screened against egg derived (E) and cell derived (C) vaccine antigen.

Clade/ Sub-Clade	Strain	Linage	BYAM			H1N1	H3N2	BVIC
		Fusion ID	PM10	BWIS141	PHU168	CAL2	SIN3	BBR3
		Parental Line	2D7	10F9	10D10	5C6	10G10	10E10
Unassigned	B/Yamagata/16/1988**^E^**		**+++**	**-**	**-**	**-**	**-**	**-**
Unassigned	B/Panama/45/1990**^E^**		**+++**	**-**	**-**	**-**	**-**	**-**
1	B/Harbin/07/1994**^E^**		**-**	**++**	**-**	**-**	**-**	**-**
Unassigned	B/Yamanashi/166/1998**^E^**		**-**	**++**	**-**	**-**	**-**	**-**
Unassigned	B/Victoria/504/2000**^E^**		**-**	**++**	**-**	**-**	**-**	**-**
1	B/Jiangsu/10/2003**^E^**		**-**	**++**	**-**	**-**	**-**	**-**
1	B/Florida/4/2006**^E,V^**		**+++**	**++**	**[+++]**	**-**	**-**	**-**
2	B/Brisbane/3/2007**^E^**		**++**	**++**	**+++**	**-**	**-**	**-**
3	B/Bangladesh/3333/2007**^E^**		**-**	**++**	**-**	**-**	**-**	**-**
3	B/England/145/2008**^E^**		**-**	**++**	**-**	**-**	**-**	**-**
3	B/Hubei Wujiagang/158/2009**^E^**		**-**	**+++**	**-**	**-**	**-**	**-**
3	B/Wisconsin/1/2010**^E,V^**		**-**	**[+++]**	**-**	**-**	**-**	**-**
2	B/Massachusetts/2/2012**^E,C,V^**		**-**	**[+++]**	**+++**	**-**	**-**	**-**
3	B/Phuket/3073/2013**^E,V^**		**-**	**+++**	**[+++]**	**-**	**-**	**-**
172Q	B/Singapore/INFTT-16-06 10/2016**^C,V^**		**-**	**++**	**[+++]**	**-**	**-**	**-**
Negative Controls	H1N1: A/Singapore/GP1908/2015**^E,V^**		**-**	**-**	**-**	**++**	**-**	**-**
	H3N2: A/Victoria/361/2011**^E,V^**		**-**	**-**	**-**	**-**	**[+++]**	**-**
	BVIC: B/Phuket/3073/2013**^E,V^**		**-**	**-**	**-**	**-**	**-**	**[+++]**

The quality of mAb reactivity was graded and represented as following: reactivity sufficient to support potency ELISA however at edge of optimal conditions outlined in methods section (++) and reactivity optimal to support a qualified potency ELISA (+++). Strains that were not screened against candidate mAbs (-). Strains selected and formulated into the commercially released Afluria™ and Flucelvax™ vaccines are identified (V). All mAbs assessed in HAI were able to neutralize both cell and egg derived viruses. Vaccine candidate strains were screened against mAbs incorporated in qualified ([+++]) and/or un-qualified ([+++]) ELISA assays.

### 3.2 Cross-reactive mAbs as qualified reagents in HA potency ELISA

Following the identification of mAbs that cross react with both egg and cell derived antigens, potency ELISAs were established and qualified for strains representing each seasonal subtype. Strain specific qualification of these assays were performed and compared to the current compendial SRID to determine intermediate precision, repeatability, accuracy, limit of quantitation, specificity and range against egg and cell derived quadrivalent formulations (Afluria™ and Flucelvax™ respectively). The application of these mAbs to determine potency demonstrated comparable qualification parameters, within the acceptance criteria, regardless of whether the antigens were egg or cell derived (**Table 5**). Overall, the ELISA method tested within parameters defined for qualification and mostly aligned with results obtained for SRID with two notable exceptions. The ELISA was significantly more sensitive in comparison to SRID with LOQs between 200 – 700 fold more sensitive. The ELISA demonstrated 100% specificity to the subtype under examination for all strains tested irrespective of whether egg or cell derived antigen was examined, whereas the SRID was specific for the influenza A strains, yet 3 out of 9 B strain assays tested failed for specificity (B Victoria 1/5 and B Yamagata 2/4). To overcome the issue of cross reactive antisera in SRID an additional step has been introduced during assay optimisation and validation where B Victoria and B Yamagata RAs are mixed and the level of overestimated potency determined. If this is above a certain threshold a subtraction is required to normalize potency.

**Table 5:** Summary of ELISA and SRID assay qualification for egg and cell derived quadravalent vaccine (Afluria™ and Flucelvax™ respectively).

Parameter	Assay	Qual-ified strains N	H3N2			H1N1			B-VICTORIA			B-YAMAGATA		
			Mean	Min	Max	Mean	Min	Max	Mean	Min	Max	Mean	Min	Max
**Repeatability** **(%CV)**	***SRID Afluria™ and Flucelvax™***	21	2.3	0.9	4.2	2.2	1.0	3.7	1.8	0.6	3.1	2.7	1.2	4.7
	***ELISA (Afluria™)***	18	4.4	3.1	5.5	4.1	3.4	6.1	4.0	1.9	6.0	8.2	4.0	12.3
	***ELISA (Flucelvax™)***	8	5.0	0.6	9.4	4.2	1.5	9.1	4.1	0.9	9.8	4.7	1.5	9.4
**IP (%CV)**	***SRID Afluria™ and Flucelvax™***	21	4.6	3.7	5.7	7.6	3.5	6.4	5.8	4.0	7.8	5.9	5.0	8.5
	***ELISA (Afluria™)***	18	3.0	2.4	3.5	4.5	0.1	11.5	4.9	3.9	5.5	7.8	4.2	11.6
	***ELISA (Flucelvax™)***	8	5.7	3.9	7.5	7.5	6.7	8.2	4.1	0.9	9.8	5.7	4.5	6.8
**Accuracy (%)**	***SRID Afluria™ and Flucelvax™***	21	83.2	92.9	107.1	103.3	96.4	110.7	96.5	90.1	106.0	95.4	88.1	104.3
	***ELISA (Afluria™)***	18	89.1	82.8	95.8	95.2	89.0	105.3	102.5	100.3	106.8	102.2	94.0	114.2
	***ELISA (Flucelvax™)***	8	101.4	90.9	117.9	106.1	98.7	113.7	101.0	89.0	113.6	101.3	89.8	117.0
**LOQ (µg HA/mL)**	***SRID Afluria™ and Flucelvax™***	21	16.0	9.2	18.9	19.0	16.5	24.9	14.7	13.4	16.2	14.7	13.0	17.0
	***ELISA (Afluria™)***	18	0.08	0.03	0.14	0.04	0.02	0.06	0.08	0.08	0.08	0.06	0.04	0.0
	***ELISA (Flucelvax™)***	8	0.04	0.01	0.06	0.03	0.03	0.03	0.02	0.02	0.03	0.06	0.06	0.06
**Specificity**	***SRID Afluria™ and Flucelvax™***	21	Specific			Specific			CR: 2/5			CR: 1/4		
	***ELISA (Afluria™)***	18	Specific			Specific			Specific			Specific		
	***ELISA (Flucelvax™)***	8	Specific			Specific			Specific			Specific		
**Range (%RP)**	***SRID Afluria™ and Flucelvax™***	21	45-170			55-144			52-156			56-145		
	***ELISA (Afluria™)***	18	25-250			25-250			25-200			25-250		
	***ELISA (Flucelvax™)***	8	50-200			50-200			50-200			50-200		

IP; Intermediate precision, LOQ; Limit Of Quantitation, CR; Cross-Reactive SRID antisera (bivalent), Specific; Does not cross react with other strains outside of nominated Sub-type and lineage (Influenza B), RP; Relative Potency. Monoclonals used in ELISA qualifications are listed in Materials section.

### 3.3 A HAI mAb capture-detection ELISA assay detects trimeric intact antigen

**Figure 1:**
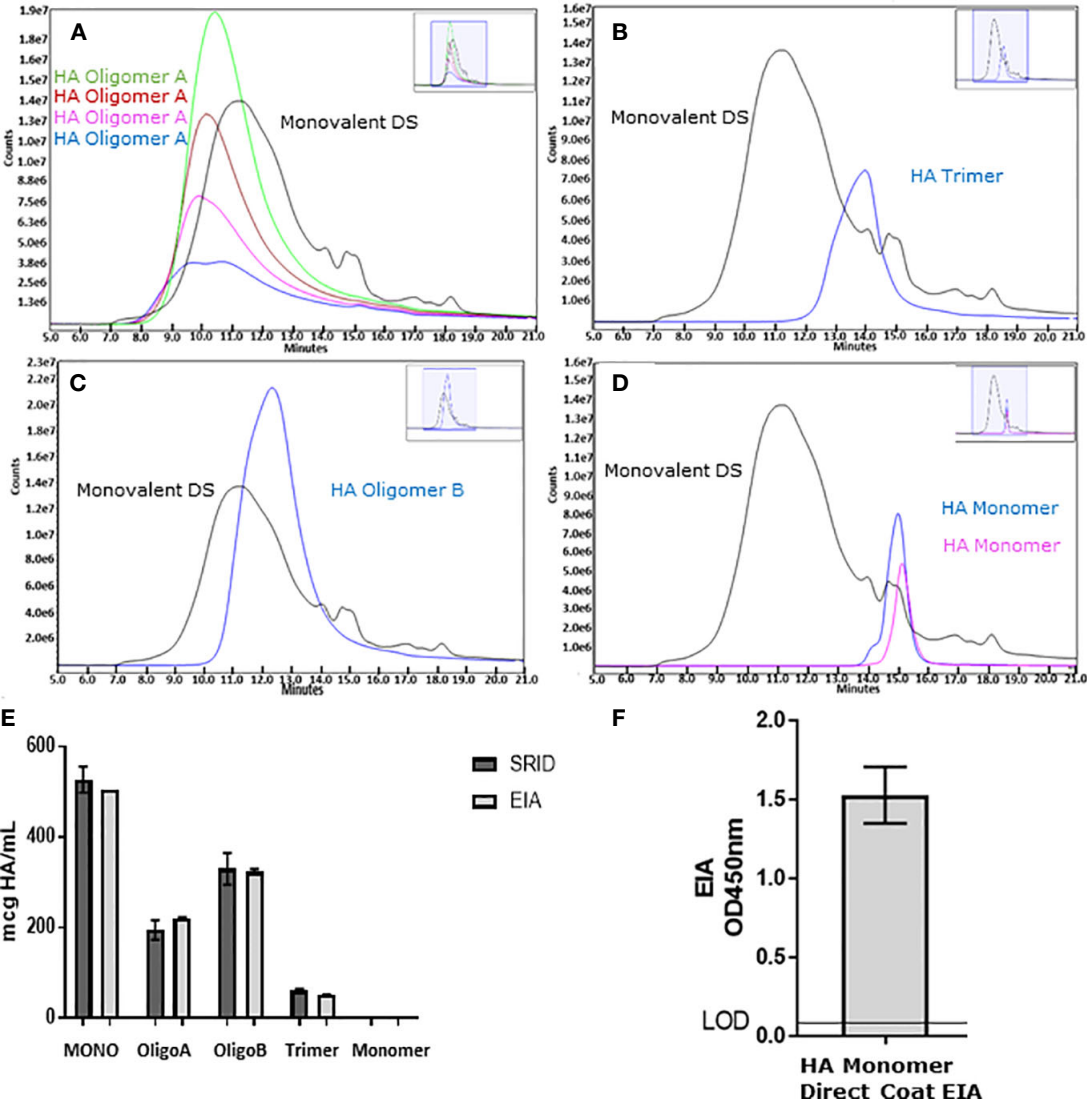
To understand the ELISA’s reactivity to different species of HA, Flucelvax^®^ Monobulk (H1N1; A/Delaware/55/2019) was separated *via* SE-UHPLC into four enriched species of HA including: HA oligomer A species >670kDa pool of sub-fractions green, red, pink and blue traces **(A)**, HA oligomer B species approximately 300kDa blue trace **(B)**, HA trimer between 200-300kDa blue trace **(C)** and HA monomer between 60-100kDa pool of sub-fractions blue and pink traces **(D)**. Fractions and Monobulk control antigen, normalised to total protein concentration, was assessed by ELISA (light grey) and SRID (dark grey) to determine HA potency **(E)**. Error bars represent standard deviation between potency replicates. Monomer HA fraction was further assessed in direct coat ELISA to verify monomeric HA sub-units were native and correctly folded **(F)**. Error bars represent standard deviation between OD450nm replicate measurements.

A vaccines efficacy is dependent upon the quality of the viral antigen(s) that are presented. For influenza vaccines, it has been well described that trimerization of the HA is required for optimal immunogenicity and best reflects the native HA of the virus (26, 27). While we previously described that our ELISA was capable of determining HA stability and correlated well with SRID (11), to further understand the antigenic relevance of this assay, HA was separated and enriched from Flucelvax™ drug substance (Monobulk) by SE-UHPLC (**Figures 1A–D**). Species of HA were separated by MW into 4 fractions designated; HA oligomers A (peak elution 10.5 minutes, >670kDa), HA oligomers B (peak elution 12.3 minutes, ~300kDa), HA trimer (peak elution 14 minutes, ~200-300kDa) and HA monomer (peak elution 15 minutes, ~60 – 100kDa), and tested in our ELISA and by SRID (**Figure 1E**). There was good correlation between the two methods detecting HA in oligomer A, oligomer B, HA trimer and HA monomer fractions (percentage similarity ELISA *vs* SRID: 96%, 113%, 98% and 84% respectively). Monomeric HA was not detected by either ELISA or SRID assay (**Figure 1E**). To confirm isolated monomeric HA was present and correctly folded, the ELISA was also performed as a direct coat assay where fractionated antigen is coated directly onto plates. This format allows mAb to bind directly to the tethered antigen circumventing mAb steric hinderance that occurs when mAbs to the same epitope are used in a sandwich format. Results confirm that HA monomer was indeed present in the 60-100kDa fraction and retains secondary structural folding (**Figure 1F**), further demonstrating the selective nature of the capture and detection ELISA for trimeric HA and higher order complexes of HA.

### 3.4 Zwittergent 3-14 pre-treatment decreases the ELISA’s sensitivity to antigenic presentation

**Figure 2:**
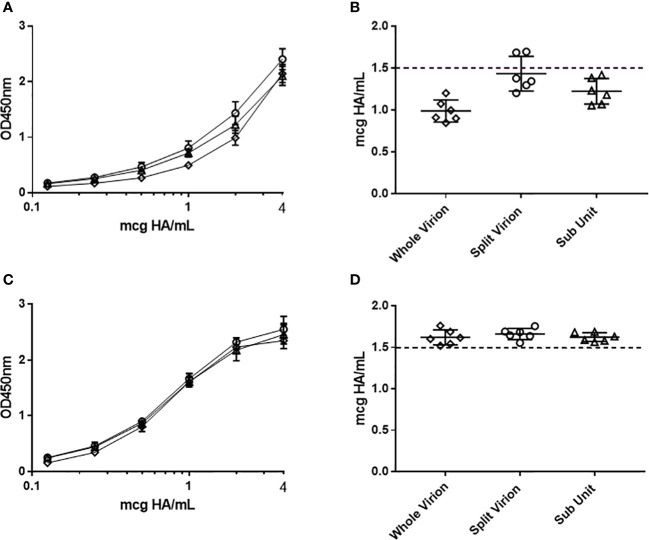
Assessment of the ELISA capture–detection method to analyse whole (diamond), split virion (circle) and sub-unit (triangle) antigen presentations. The assay was performed without **(A, B)** and with **(C, D)** sample pre-treatment using 0.5%(w/v) zwittergent 3-14 as described. Serial titration of antigen with average OD450nm responses are pictured **(A, C)** together with corresponding potency calculated from RA **(B, D)**. Error bars indicate standard deviation between replicate OD450nm raw data **(A, C)** and standard deviation of corresponding ELISA potency replicates **(B, D)**.

To understand the accuracy of the ELISA across platform and manufacturing stages, different presentations of antigen were assessed. Purified egg derived whole virion, detergent disrupted (taurodeoxycholate) egg derived split virion and purified cell derived sub-unit (HA and NA proteins) antigen of H1N1 strain A/Singapore/GP1908/2015 was standardized according to HA content by non-native SDS-PAGE densitometry (28) and assessed for HA potency by ELISA (**Figure 2**). In the absence of a pre-treatment step, there was high variability in potency by ELISA (CV = 16.7%, **Figures 2A, B**). To equilibrate the presentation of these antigens, we examined the detergent Zwittergent 3-14 as well as a range of other excipients, non-ionic and zwitterionic detergents (data not shown). Of the solutions studied, Zwittergent 3-14 at a concentration of 0.5% v/v performed the best in normalizing the presentation of HA across the different sample types, aligning the reported concentrations closer to the expected target formulation of 1.5 mcg HA/mL (1.5 mcg HA/mL was targeted because we were investigating dose sparing formulations) and reducing the variability across sample type (CV = 3.5%, **Figures 2C, D**).

### 3.5 ELISA has stability indicating properties similar to those of SRID

**Figure 3:**
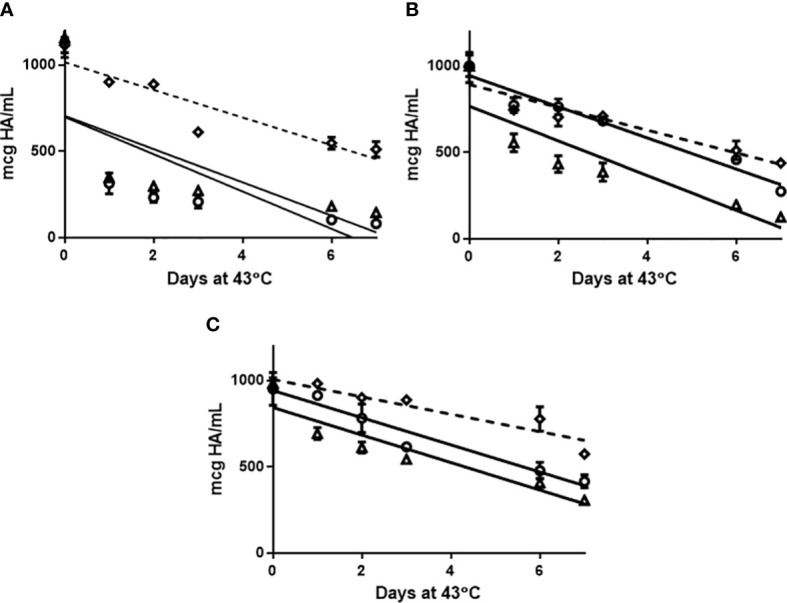
Afluria^®^ drug substance representing 2015 NH strains: A/California/07/2009 H1N1 **(A)**, A/South Australia/55/2014 H3N2 **(B)** and B/Phuket/3073/2013 B Yamagata **(C)** were held at 43°C for 7 days. Aliquots were removed at days: 0, 1, 2, 3, 6 and 7 and assessed for potency by monoclonal ELISA (circle), SRID (triangle) and polyclonal ELISA (Diamond). Slope comparison determined by linear regression modelling was plotted (slope full line: mAb ELISA and SRID, slope broken/dash line: pAb ELISA) and assessed in results section. Error bars represent standard deviation between assay replicates.

The ELISA was assessed to determine if it was stability indicating (**Figure 3**). An accelerated stability study was performed and indicated the ELISA and SRID were comparable for detecting the rate of degradation of antigens (ELISA: mean +/-SD: -92.3 +/- 14.6), SRID: mean+/-SD: -92.0 +/- 10.6). The use of a zwittergent detergent analogous to SRID prompted us to examine replacing the detection reagent following mAb capture with an existing polyclonal antibody used in the SRID assay. Given the SRID antisera is polyclonal in nature, binding to multiple epitopes of HA with a range of specificities, our hypothesis was that this capture-detection format would measure both trimeric and monomeric HA as well as partially unfolded antigen. Performance of the pAb ELISA in the accelerated stability study, demonstrated that linear regression (slope) was less pronounced than the mAb ELISA and SRID (mean+/-SD: -65.3 +/- 15.0). Furthermore, there was no statistical difference in the rate of degradation measured by mAb ELISA and SRID whereas differences statistically comparing the rate of degradation measured by pAb ELISA *vs*. mAb ELISA and SRID was reported (Student Test: mAb ELISA *vs* SRID p=0.926; mAb *vs* poly ELISA p=0.020, SRID *vs* poly ELISA p=0.049). Thus, the use of the polyclonal reagents in the ELISA may compromise the required stability indicating quality of a vaccine with respect to potency.

### 3.6 Monoclonal potency ELISA has broad application across vaccine platforms and against different forms of HA

To further demonstrate the broad application of our ELISA, influenza HA antigen representing a range of licensed and research grade manufacturing platforms, hosts and vectors were analyzed to exhibit the sensitivity of our TORA184.9G2 and CAL2.5C6 mAb clones by determining the LOD and LOQ of candidate H1N1 strains produced *via* alternative expression vectors and/or alternative manufacturing platforms (**Table 6**). The TORA184.9G2 mAb was selected as it cross-reacted with H1N1 strains: A/Victoria/2454/2019 (sourced to assess HA derived from embryonated chicken eggs from a selection of typical manufacturing stages and platforms; BPL and formalin inactivated whole virus, monovalent and quadravalent formulated split virus and purified surface antigen quadravalent formulation), A/Wisconsin/588/2019 (sourced to assess the baculovirus expressed recombinant HA product FluBlok^®^) and A/California/07/2009 (sourced to assess full-length HA expressed from cDNA in a baculovirus host). The CAL2.5C6 mAb was utilized for the A/Brisbane/02/2018 strain (sourced to assess HA1expressed from cDNA in both E.coli and HEK293 cell hosts) as the TORA184.9G2 mAb did not cross-react with H1N1 sub-clade 6b1.A/183P-1 (**Table 2**). The H1N1 strains used in this study were selected based on availability at the time of the study. The analysis indicated, 11 out of 13 alternative presentations of HA were detected using the TORA184.9G2 mAb alone, demonstrating strong affinity to HA with LODs ranging between 0.5 - 125 ng/ml of HA and good accuracy at low concentrations of HA with LOQs ranging between 20 - 400 ng/mL of HA. The A/Brisbane/02/2018 HA1 protein, recombinantly expressed from cDNA in E.coli and HEK293 cell culture did not react in our capture detection ELISA therefore LOQ was not perused (29).While LOQ was unable to be determined *via* capture detection ELISA for recombinantly expressed HA1 we were still able to demonstrate the reactivity of CAL2.5C6 mAb against HEK293 and E.coli expressed HA1 by directly binding (titrating) antigen to immuno-absorbent plates and detecting with mAb. LODs were calculated for HEK293 and E.coli expressed HA1 to be 250 ng/mL of HA and 625 ng/mL of HA respectively.

**Table 6:** Compatibility of ELISA with various influenza vaccine platforms and HA expression vectors.

Host	HA Presentation and Manufacturing Platform	Strain	Monoclonal Antibody	LOD	LOQ
				ng HA/mL	
**Chicken embryonated egg**	Whole, infectious virus – Afluria^®^	A/Victoria/2454/2019	TORA184.9G2	0.5	400
	Whole, BPL Inactivated – Afluria^®^	A/Victoria/2454/2019	TORA184.9G2	10	400
	Whole, Formalin Inactivated – NIBSC	A/Victoria/2454/2019	TORA184.9G2	30	400
	Split Virion Monovalent – Afluria^®^	A/Victoria/2454/2019	TORA184.9G2	20	400
	Split Virion Quadravalent – Afluria^®^	A/Victoria/2454/2019	TORA184.9G2	20	400
	Surface Antigen, Quadrivalent – Fluad^®^	A/Victoria/2454/2019	TORA184.9G2	20	400
**Cell, HEK293**	cDNA recombinant HA1, his-tagged.	A/Brisbane/02/2018	CAL2.5C6	250*	BLD
**Cell, MDCK**	Whole, BPL Inactivated – CBER	A/Delaware/55/2019	TORA184.9G2	63	400
	Surface Antigen, Monovalent – Flucelvax^®^	A/Delaware/55/2019	TORA184.9G2	125	400
	Surface Antigen, Quadravalent – Flucelvax^®^	A/Delaware/55/2019	TORA184.9G2	125	400
**E.coli**	cDNA, recombinant HA1, his-tagged.	A/Brisbane/02/2018	CAL2.5C6	625*	BLD
**Baculovirus - Insect Cells**	Recombinant – FluBlok^®^	A/Wisconsin/588/2019	TORA184.9G2	1	20
	cDNA recombinant HA	A/California/07/2009	TORA184.9G2	10	100

*Not reactive in capture detection format; limit of detection (LOD) determined from direct antigen coated method for these samples only. LOD is an estimate in these cases as direct-coating efficiency impacts accuracy. Limit of quantitation (LOQ) was not pursued in this format. Below limit of detection (BLD). The sensitivity of H1N1 ELISA mAbs: TORA184.9G2 and CAL2.5C6 to these samples was demonstrated by measuring the ELISAs Limit of detection (LOD) and Limit of Quantitation (LOQ) in accordance with qualification method described.

### 3.7 The ELISA potency method is compatible with commonly used vaccine adjuvants

In order to increase the immunogenicity of vaccines in the immunocompromised such as the elderly as well as dose-sparing in a pandemic setting for poorly immunogenic antigens like H5N1, adjuvants are commonly used in antigen vaccines to enhance induction of neutralizing responses (30–37). To test the utility of our ELISA potency method we analyzed vaccine formulations containing two commonly used vaccine adjuvants; MF59™, Seqirus’s squalene based oil-in-water emulsion (38) and aluminum phosphate (39). The accuracy of the ELISA to determine potency in these formulations were compared to potencies derived by SRID (**Table 7**). The inclusion of MF59™ into the quadrivalent formulation had no notable impact on the accuracy of the ELISA or SRID to measure HA potency even at the highest concentration of squalene examined (>50% higher than standard amount) with observed *vs*. expected (calculated DS dilution based on formulation target) results within 15% difference. However, analysis of aluminum adjuvanted pandemic H5N1 demonstrated a notable difference between the ELISA and SRID assays. Where ELISA was unaffected by the adjuvant (accuracy 101% observed *vs* expected); the SRID was inhibited by the aluminum phosphate with an accuracy of 77% (observed *vs* expected).

**Table 7:** Compatibility of ELISA with adjuvants: MF59 at standard concentration (>7.5mg squalene/15µg HA), 20% higher concentration then standard (>9.2mg squalene/15µg HA), 50% higher concentration then standard (>11.8mg squalene/15µg HA) and ALPO4 at typical target concentration.

Formulation Matrix	MF59 Adjuvant (>7.5 mg squalene per 15 mcg HA)	MF59 Adjuvant (>9.2 mg squalene per 15 mcg HA)	MF59 Adjuvant (>11.8 mg squalene per 15 mcg HA)	AlPO_4_ Adjuvant (>0.5 mg Al3+ per 41 mcg HA)
Representative Sub-type	H3N2	H3N2	H3N2	H5N1
Presentation	Quadrivalent	Quadrivalent	Quadrivalent	Monovalent
Representative Strain	A/Delaware/39/ 2019	A/Delaware/39/ 2019	A/Delaware/39/ 2019	A/Egypt/NO3072/ 2010
Expected HA Potency (mcg/mL)	38.0	30.4	19.0	82.0
ELISA HA Potency (mcg/mL)	34.6	27.1	17.0	83.1
SRID HA Potency (mcg/mL)	34.6	27.7	17.3	63.0
ELISA Obs/Exp (%)	91%	89%	90%	101%
SRID Obs/Exp (%)	91%	91%	91%	77%

## 4 Discussion

The regulatory requirement of influenza vaccines is currently dependent upon the antigenicity and potency of HA within a formulated dose. For Influenza vaccine, potency is determined using an immuno-analytic surrogate to measure HA content quantified against a standardized reference antigen. This measurement is not only necessary for vaccine formulation and release but required to ensure vaccine stability and shelf-life. Since 1978 the SRID assay has been globally accepted as the dominant method for potency determination and release of inactivated influenza vaccines (40). While the SRID has served its purpose well for many decades there are limitations to the approach which are becoming more apparent. Most critically, SRID’s implementation is slow due to the requirement of considerable amounts of strain-specific reagents for each strain within the multivalent vaccine formulations, the production of which takes a significant time period usually around two months. The antiserum is made by immunizing sheep with highly pure haemagglutinin whilst the antigen reference must be strain and often host matched to that used in the manufacturer’s vaccine. Given the sheer scale and purity required of these materials used to produce SRID reagents, they generally can only be supplied by the large-scale influenza vaccine manufacturers. The co-operative nature of reagent production, calibration and need for rapid production if the critical window for receiving the vaccine is to be met, makes for a complex and rigid timetable (2). Earlier availability of vaccines would significantly enhance their overall benefit in the case of a pandemic. It has been noted in the case of the 2009-2010 H1N1pdm09 influenza pandemic, more than 2,000 deaths in the US may have been prevented if not for the delay in availability of vaccines at the epidemic’s onset (41, 42).

In this report, we extend on our previous findings with the identification of multiple mAbs appropriate for a capture and detection potency ELISA to measure HA. The sub-type specific mAbs can be rapidly optimised, qualified and utilized for multiple strains, across clades/sub-clades, for an average of 6 seasons (3 years) of influenza drift, decreasing the requirement to update reagents for each strain change which is commonly done every 6 months. Some mAbs such as the SIN178.10G10 line specific to H3N2s, had notably superior cross-reactive longevity relative to other mAbs. Retrospectively, this mAb could have been utilized in qualified ELISA’s for greater than 13 years of H3N2 drift including the current 2022 Southern Hemisphere seasons vaccine. An effort to map predicted binding locations of our mAbs is currently underway using a combination of bio-physical and in-silica methodology (data not shown). Interim findings suggest an interesting pattern of binding localized within epitope region D of the Influenza A HA1 sub-unit (43–45). While it is generally acknowledged the implementation of SRID assay into manufacturing QC laboratories from WHO’s announcement takes as long as 4 months (1, 2, 46), we have been able to achieve this in parallel studies in under 6 weeks using our cross-reactive mAbs. This timeline includes a comprehensive qualification package focused specifically on introduction of new stains into the vaccine. The performance of the ELISA against both egg and cell derived virus was comparable to the SRID with two advantageous exceptions. We were able to demonstrate complete sub-type specificity and, importantly, linage specificity between Influenza B Victoria and Yamagata lineages, something the SRID was unable to achieve for 33% of Influenza B strains tested.

A recent publication has discussed the possibility of including a third Influenza A strain into the quadravalent vaccine noting difficulties at strain selection due to sub-clade divergence, primarily within the H3N2 seasonal sub-type (47). The format of our ELISA together with recent advances in our hybridoma methodology has allowed selection of specific mAbs to highly targeted epitopes giving us the flexibility to begin producing a library of clade/sub-clade specific mAbs within the H1N1 and H3N2 seasonal sub-types, something that is unlikely to be achieved with the SRID assay. It is generally accepted that neutralizing antibodies are conformation-specific, and that complete HA antigenicity and superior immunogenicity requires subtle conformational effects that accompany HA trimerization (27). A study by Wei et al. (2008) compared the immunogenicity of mammalian cell-expressed HA oligomers, trimers, and monomers in mice. Monomeric HA failed to induce significant neutralizing antibodies against H5N1 whereas high-molecular-weight oligomers and HA trimer elicited the highest titers of neutralizing antibody (26). By fractionating HA rich Monobulk drug substance into fractions of HA monomer, trimer and higher order oligomeric species of HA, we were able to demonstrate the preferential nature of our potency ELISA to detect HA trimer and HA oligomers only and not detect immuno-irrelevant HA monomer antigen. The importance of using HA monoclonals in the prescribed ELISA format was also highlighted in accelerated stability experiments where data was compared with data from a polyclonal antisera (pAb) ELISA format. Results demonstrated a greater degree of stability indication, comparable to SRID, for our mAb ELISA relative to the pAb ELISA format which had significantly shallower slopes and reported higher potencies at each time-point post time 0. It is likely the pAb EIA is detecting both native and denatured HA relative to mAb EIA and SRID which are detecting de-natured HA at a significantly lower rate, if at all.

The emergence of SARS-CoV-2 pandemic in 2019 has once again put vaccination in the spotlight globally. The renewed interest, and financial stimulus, has led to a boon in vaccine research and development programs, including in the area of influenza vaccines. Connected with the latest technology and science, vaccine platforms such as mRNA, Virus Like Particles (VLPs), recombinant expression vectors and cell culture are being utilized in clinical trials and vaccine registration submissions at increasing rates. Given the specific nature of selected antibodies in our ELISA we wanted to ensure the assay was robust and not limited to a single manufacturing platform. We adjusted our screening regime to include the requirement to react with both egg and cell-derived antigen after immunizing mice with egg-based antigen. In all cases to date, we were able to identify mAbs, representing seasonal quadravalent viruses, that could detect both egg and cell antigens by HAI. We further demonstrated using H1N1 as a test case, that our ELISA could be used for vaccines with HA derived from a variety of commonly used hosts including; embryonated eggs, E.coli, MDCK cells and baculovirus cells as well as HA antigen from various platforms and manufacturing stages including: whole virus (non-inactivated and inactivated with BPL and formalin), split virion (TDOC detergent disrupted), surface antigen purified/sub-unit (CTAB detergent disrupted) and recombinant protein expressed from a cDNA vector.

While the sensitivity of SRID, which is generally accepted to be between 5-15 µg/mL HA (2, 48), is suitable for current vaccines formulated at 15 µg HA per dose (30 µg/mL HA in a 0.5ml dose), with advances in adjuvants and vaccine technology, dose sparing of HA below the LOQ of SRID will become feasible. This is of particular interest in the area of pandemic preparedness (2, 48, 49). Determining the LOD and LOQ of representative strains, we demonstrated the ability to accurately quantitate at nanogram concentrations of HA per mL for each component of the seasonal quadravalent vaccine. In a separate study, we further demonstrated high sensitivity of quantitation for vaccines from a variety of different hosts, platforms and manufacturing stages. Of particular note, our TORA184.9G2 mAb had a notably high affinity and accuracy for baculovirus expressed HA, especially testing the FluBlok^®^ quadravalent vaccine product where HA could be detected above background at a concentration of 1 ng/mL and could be accurately quantitated at a concentration of 20 ng/mL, which is 250 fold lower in concentration than the typically quoted LOQ of SRID (2, 48). Recombinant HA1 expressed in E. Coli and HEK293 could be detected by direct-coat ELISA but not using our capture and detection ELISA. Had we procured full length cDNA expressed HA, as was achieved in the baculovirus cell host, we are confident the capture-detection ELISA method would have functioned and an LOQ measurement achieved. While purchasing HA1 only was an unfortunate oversight on our behalf, this result further demonstrates the important stability indicating properties of our ELISA. The recombinantly expressed HA1 would be truncated in nature. While we cannot rule-out steric hindrance as the reason these where not detected in our capture/detection ELISA, the truncated rHA1 proteins are also likely monomeric as the HA2 sub-unit is structurally required for non-covalent association of HA monomers to form stabilized trimer (29). While we were unable to acquire VLP formulations containing HA, we have previously demonstrated sensitive reactivity of our mAbs generated against SARS-CoV-2 Spike protein in ELISA testing SARS-CoV-2 VLP formulations (data not shown). These mAbs were produced using the same hybridoma methodology and screening protocols used for our influenza mAbs.

Given our mAbs are HA inhibiting, thus likely to be conformation dependent, we were aware that mAb affinity and avidity would likely be affected by differences in accessibility, oligomerization, aggregation and post-translational modification of HA from different platforms and hosts. We demonstrated that when HA antigen from different hosts, platform or processing stages were formulated to equivalent levels of HA using a destructive HA content approach, the potencies determined by ELISA differed. We were able to develop a pre-treatment method similar to that of SRID using a zwitterionic detergent and demonstrate successful normalization of HA content level present between whole (egg derived), split (egg derived) and subunit (cell derived) antigens. Influenza vaccines can also differ by the addition of adjuvants such as MF59 and aluminum phosphate included to enhance the immunogenicity of the vaccine (30–37). Unlike the SRID, which under-reported potency in the presence of aluminum, the ELISA was not affected by aluminum or MF59 type adjuvants broadening its applicability to formulations containing these two popular adjuvants.

In this investigation, we focused on measurement of vaccine potency relative to the content of immuno-relevant HA antigen as this is the requirement for Influenza vaccine registration world-wide, however Neuraminidase (NA) also plays a key role in serological protection against seasonal and pandemic outbreaks (50). Studies have demonstrated that NA-inhibiting antibodies contribute to overall immunity (51–53) and were associated with fewer symptoms, reduced symptom severity score and reduced duration of symptoms and shedding (54). Quantitative assessment of NA using a monoclonal-based ELISA assay is feasible and few have reported good correlation between NA concentration and immunogenicity using this approach (55–57). We have also produced a library of NA-specific mAbs which are used routinely in our screening and characterisation studies including: an N1-specific mAb that cross reacts with H1N1 and H5N1 candidate strains (CFNA150.6B7), an N3-specific mAb (OHIO187.9B11) and a mAb raised against a conserved epitope of Influenza B viruses that cross-reacts with strains from both B Victoria and B Yamagata lineages (NAB147.6G3). While we have demonstrated proof of principle that NAB147.6G3 mAb works in our capture and detection ELISA format and can be quantitative utilizing a purified NA standard, we are yet to demonstrate accuracy, precision and stability indication.

It is common knowledge within the influenza vaccine community that implementation of SRID is the primary rate limiting step to release of vaccine. We have demonstrated extensively the ELISA’s cross-reactive properties between strains of a sub-type, reduces the need for matched reagents for every vaccine strain update. This, together with the need for lower quantities and concentrations of reference antigen, reduces the lead time from announcement of strain prototype to the implementation of a validated assay, considerably. We estimate a mAb cross-reactive strain could be implemented into a GMP laboratory for vaccine release in as little as 6 weeks from WHO announcement, where the average time taken for SRID implementation is 12-14 weeks (2, 9, 10). However, in-order for this strategy to be successful, pro-active mAb screening/characterisation, linked with thorough strain surveillance are vital. In the case a novel clade or sub-type emerges where no reactive mAb is available the time required to implement ELISA extends from 6 weeks to 12+ weeks, thus negating the assays primary benefit over SRID. While we have made inroads in advancing our mouse immunization strategy to produce mAbs to targeted epitopes, thus reducing overall time to generate qualified ELISAs against novel strains, our overall objective is to have a library of mAbs that not only cover currently circulating seasonal and pandemic strains, but also mAbs against new and emerging, clades, sub-clades and pandemic sub-types, so that the risk of not having an available assay is minimal. With the development of powerful algorithms and advances in bioinformatics, together with new and improved methods to produce recombinant HA with targeted mutations, it may soon possible to generate mAbs against predicted clades that have not yet emerged in nature. As this study was for investigational purposes, reference antigens used in our ELISA were developed and calibrated within our laboratory using biophysical methods, independent of Essential Regulatory Laboratories (ERLs). While we were able to demonstrate good correlation with SRID using this approach, we acknowledge in a GMP vaccine release scenario that reference antigens, whether homogenous or heterologous in nature, would require ERL calibration and oversight as is required with SRID.

In summary, our analysis demonstrates that the HA potency ELISA described is applicable, with the inclusion of a pre-treatment step, to accurately detect the potency and antigen stability in a wide range of influenza vaccine manufacturing platforms, host organisms and formulation matrices, as long as the material is in its native, trimeric form considered the most immunogenic thus suitable for vaccination. We have previously demonstrated that this assay correlates well with the SRID assay (11) and have performed this assay in parallel with SRID in multiple clinical trials (manuscript in preparation). Further, we are confident with recent advances in the immunogen we use for producing mouse hybridomas, we will be able to produce targeted mAbs specific to strains of quadrivalent influenza vaccines containing two viruses from the same influenza A subtype, e.g. two H3N2 strains, a H1N1 strain and a single B/Victoria-lineage strain as it appears that B/Yamagata-lineage viruses may have become extinct and may not be required in future influenza vaccines (58). We believe that the ELISA is appropriate for the majority of platforms that are currently available for licensed influenza vaccines as well as new platforms on the horizon and should be considered for global application as an alternative to be run in parallel with SRID in the short term, and if successful, one day replace SRID.

## Data availability statement

The raw data supporting the conclusions of this article will be made available by the authors, without undue reservation.

## Author contributions

Conceptualization and study design JB, SR, IB and KV. Laboratory work JB and KV. Data analysis JB, KV, KL, YZ and SR. Writing and Editing JB, KV, KL and SR. All authors contributed to the article and approved the submitted version.
